# Clinical Characteristics and Factors Associated With Hypertension in 205 Hospitalized Children: A Single-Center Study in Southwest China

**DOI:** 10.3389/fped.2021.620158

**Published:** 2021-04-07

**Authors:** Zhiyong Yang, Yanyun Huang, Yan Qin, Yusheng Pang

**Affiliations:** Department of Pediatrics, The First Affiliated Hospital of Guangxi Medical University, Nanning, China

**Keywords:** primary hypertension, secondary hypertension, children, target organ damage, associated factors

## Abstract

**Objective:** The aim of this study was to investigate the clinical characteristics and factors associated with pediatric hypertension and target organ damage (TOD).

**Methods:** We retrospectively reviewed clinical data from 205 children with hypertension treated in our hospital from 2007 to 2018. The patients were classified based on the type of hypertension (primary, secondary) and presence of TOD (heart, brain, retina). Logistic regression analysis was performed to identify the factors independently associated with hypertension and TOD.

**Results:** There were 107 males, 97 females, and one intersex in this study, with an age range of 0.1–17.9 years. Majority of cases (177, 86.3%) had secondary hypertension, while 13.7% had primary hypertension. The most frequent cause of secondary hypertension was renal disease (59.32%). Elevated serum creatinine level (odds ratio [OR] = 7.22, 95% confidence interval [95% CI] = 1.6–32.62, *P* = 0.01), blood urea nitrogen (OR = 6.33, 95% CI = 1.81–22.19, *P* = 0.004), serum uric acid level (OR = 3.66, 95% CI = 1.20–11.22, *P* = 0.023), and albuminuria (OR = 3.72, 95% CI = 1.50–9.26, *P* = 0.005) were independently associated with secondary hypertension. Elevated serum uric acid and blood urea nitrogen levels were associated with left ventricular hypertrophy (OR = 6.638, 95% CI = 1.349–32.657, *P* = 0.02) and hypertensive encephalopathy (OR = 4.384, 95% CI = 1.148–16.746, *P* = 0.031), respectively. Triglyceride level correlated with hypertensive retinopathy (*P* = 0.001).

**Conclusion:** Pediatric hypertension was most often secondary, with renal disease as the leading cause. Elevated levels of serum uric acid, blood urea nitrogen, serum creatinine, and albuminuria may indicate secondary hypertension in childhood. Elevated serum uric acid, blood urea nitrogen, and triglyceride levels were associated with left ventricular hypertrophy, hypertensive encephalopathy, and hypertensive retinopathy, respectively.

## Introduction

Hypertension, an increase in systemic arterial pressure, is a major public health issue worldwide (1). It is a key contributor to cardiovascular and cerebrovascular diseases, causing over 7 million deaths globally, and accounts for 4.5% of the global disease burden (2). Although cardiovascular events rarely occur in children, pediatric hypertension has been identified as a predictor of adult hypertension and a potential risk factor for cardiovascular disease and mortality (3). Data on blood pressure (BP) tracking from childhood to adulthood have demonstrated that elevated BP in childhood increases the risk of developing hypertension in adulthood (4, 5). Hence, early detection of childhood hypertension and timely intervention may reduce the risk of cardiovascular disease in adults.

Although pediatric hypertension used to be considered as a rare disease, epidemiological studies have shown its growing prevalence, with a relative increase rate of 75–79% from 2000 to 2015 globally (6). Prevalence estimates are variable among different countries because of different populations and standards. Most studies have estimated the prevalence of pediatric hypertension in the range between 2 and 5% (7, 8), which likely increases with age.

Pediatric hypertension is mostly asymptomatic (9). However, target organ damage (TOD) has been identified at the initial diagnosis of hypertension in children (9). About 40% of newly diagnosed hypertensive children presented with left ventricular hypertrophy (LVH) (9). Since TOD may occur even in children with pre-hypertension or with only slightly elevated BP, any elevation of BP can be detrimental (10, 11). Hence, early identification and intervention may be warranted in hypertensive children. Previous studies have shown that uric acid, high salt intake, inflammatory factors, BP variability, body mass index (BMI) Z score, and central systolic BP are associated with TOD in hypertension (12–15). However, those studies were pre-dominantly conducted in adults. There remains limited data on the factors associated with TOD in children. Thus, we conducted a single-center study in southwest China. In this study, we retrospectively analyzed the clinical characteristics and factors associated with hypertension and TOD in hospitalized children with hypertension to provide a reference for the diagnosis and treatment of pediatric hypertension.

## Patients and Methods

### Study Subjects

Consecutive patients aged ≤ 18 years with hypertension admitted to the First Affiliated Hospital of Guangxi Medical University from January 2007 to December 2018 were included in the current study. The exclusion criteria encompassed transient hypertension, perioperative hypertension, and patients with insufficient data in their clinical records. This study was approved by the Ethics Committee of the First Affiliated Hospital of Guangxi Medical University [Nanning, China, NO. 2021(KY-ABCDE-022)].

### Definition

In children between 1 and 13 years of age, hypertension was defined as mean systolic blood pressure (SBP) and/or diastolic blood pressure (DBP) ≥95th percentile for the respective sex, age, and height, or ≥130/80 mmHg measured on more than three occasions (whichever was lower). Stage 1 hypertension was defined as SBP and/or DBP between 95th percentile and 95th percentile + 12 mmHg, or 130/80–139/89 mmHg (whichever was lower). Stage 2 hypertension was defined as SBP and/or DBP higher than the 95th percentile + 12 mmHg, or higher than 140/90 mmHg (whichever was lower) (16). In children aged 13 years and older, hypertension was defined as BP ≥130/80 mmHg. Stage 1 hypertension was defined as BP between 130/80 and 139/89 mmHg, and stage 2 hypertension was defined as BP ≥140/90 mmHg (16). Secondary hypertension was diagnosed when the etiology of high BP was clearly identified, in contrast to primary hypertension. LVH was defined as left ventricular mass index >51 g/m^2.7^ according to echocardiography (17). Hypertensive retinopathy was classified into four grades based on the Keith-Wagener classification (18): grade I (mild narrowing of the retinal arterioles), grade II (moderate retinal arteriolar stenosis or arteriovenous nicking), grade III (vascular lesions with cotton-like exudate or hemorrhages), and grade IV (optic disc edema). Hypertensive encephalopathy was defined as BP >150–160/100–110 mmHg with more than one neurological symptom out of the following: vomiting, diplopia, severe headache, transient blindness, convulsions, or loss of consciousness.

### Study Methods

All data were obtained from electronic medical records. We collected general information including age, sex, height, weight, and BMI. We recorded the results of laboratory examinations, including urinalysis, levels of blood urea nitrogen (BUN), serum uric acid (SUc), serum creatinine (SCr), low-density lipoprotein (LDL), triglycerides (TG), total cholesterol (TC), C-reactive protein (CRP), alanine aminotransferase (ALT), aspartate aminotransferase (AST), aldosterone, rennin, and angiotensin. Electrocardiogram and imaging examinations, such as echocardiography, chest X-ray, craniocerebral computed tomography (CT), and magnetic resonance imaging (MRI), were conducted. The patients were classified into the LVH group and non-LVH group, hypertensive retinopathy group and non-hypertensive retinopathy group, and hypertensive encephalopathy group and non-hypertensive encephalopathy group, based on the presence or absence of specific TOD of the heart, retina, and brain, respectively. The participants were also classified into five groups based on age (<1 year, 1–3 years, 3–7 years, 7–12 years, ≥12 years).

### Statistical Analysis

Data analysis was completed using SPSS software (Version 22.0 for Windows, SPSS, Inc., Chicago, Illinois). Categorical variables are presented with frequencies and percentiles, and continuous variables with mean values and standard deviations. Continuous variables were compared between two groups using Student's *t-*test (for normal distribution) or Wilcoxon rank sum test (for skewed distribution). Normality of distribution was tested using the Shapiro–Wilk normality test. The chi-square test or Fisher's exact probability test was used for comparing categorical variables. The factors associated with hypertension and TOD were evaluated by logistic regression analysis. The results are presented as odds ratios (OR) and 95% confidence intervals (95% CI). The variables with a *P*-value < 0.05 in the univariate analysis were included in the multivariate logistic regression analysis. For all comparisons, *P* < 0.05 was considered statistically significant.

## Results

### General Characteristics

A total of 205 consecutive patients were included in this study, including 107 males, 97 females, and one intersex, with an age range from 0.1 to 17.9 years. Average BMI was 17.33 ± 3.81 kg/m^2^. Only 28 cases (13.7%) showed primary hypertension, while 177 (86.3%) children had secondary hypertension. In both the primary hypertension group and secondary hypertension group, the highest proportion of subjects belonged to the group aged ≥12 years. The general characteristics and age distribution of the patients are shown in Table 1.

**Table 1:** General characteristics and age distribution of hypertensive children.

**Characteristics**	**Primary hypertension group**	**Secondary hypertension group**	***P*-value**
Total numbers (n, %)	28 (13.66)	177 (86.34)	
Sex, male (n, %)	18 (64.28)	89 (50.28)	0.168
Age groups (n, %)			0.501
<1 years	2 (7.14)	8 (4.52)	
1–3 years	2 (7.14)	16 (9.04)	
3–7 years	1 (3.57)	27 (15.25)	
7–12 years	10 (35.71)	59 (33.33)	
≥12 years	13 (46.43)	67 (37.85)	
Hypertension classification (n, %)			0.545
Stage 1 hypertension	3 (10.7)	10 (5.6)	
Stage 2 hypertension	25 (89.3)	167 (94.4)	
BMI (kg/m^2^)	17.81 ± 5.36	17.25 ± 3.52	0.598

### Secondary Causes of Hypertension

Renal disorders were the most frequent cause of secondary hypertension in the current study (Supplementary Materials). Among 177 subjects with secondary hypertension, renal disorders were detected in 59.32% of patients (including renal parenchymal disease in 55.37% and renovascular disease in 3.95%). Drug-induced hypertension was recorded in 29.94% of patients, whereas, other causes of secondary hypertension were rare (cardiovascular disease in 3.39%, endocrine disorders in 3.39%, nervous system disorders in 3.39%, and unclassified causes in 0.56% cases).

### Factors Associated With Secondary Hypertension

To investigate the factors associated with secondary hypertension, we compared the primary and secondary hypertension groups (Table 2). The proportions of elevated SCr, elevated BUN, elevated SUc, and albuminuria were significantly greater in the secondary hypertension group than in the primary hypertension group (*P* < 0.05), while no significant inter-group differences were found in the other laboratory results, sex, and BMI. Multivariate assessment by logistic regression analysis revealed that the four variables remained independently associated with secondary hypertension. Elevated SCr was the strongest predictor of secondary hypertension (OR = 7.22, 95% CI = 1.60–32.62, *P* = 0.01), followed by elevated BUN (OR = 6.33, 95% CI = 1.81–22.19, *P* = 0.004), albuminuria (OR = 3.72, 95% CI = 1.50–9.26, *P* = 0.005), and elevated SUc (OR = 3.66, 95% CI = 1.20–11.22, *P* = 0.023).

**Table 2:** Variables of primary and secondary hypertension.

**Parameters**	**Primary group**	**Secondary group**	****χ^2^/Z****	***P*-value**
Sex, male (%)	64.28	50.28	3.614	0.816
BMI (kg/m^2^)	17.8 ± 5.36	17.25 ± 3.52	0.533	0.598
Albuminuria (%)^*^	25.9	56.6	8.809	0.003
Elevated SUc (%)^*^	17.4	43.5	5.748	0.017
Elevated BUN (%)^*^	13.6	50	10.387	0.001
Elevated SCr (%)^*^	11.1	47.4	8.558	0.003
Elevated TG (%)^*^	38.5	42.5	0.081	0.776
Elevated TC (%)^*^	30.8	46.3	1.151	0.283
Elevated LDL (%)^*^	61.5	37.4	0.006	0.94
Elevated AST (%)^*^	22.7	28.4	0.313	0.576
Elevated ALT (%)^*^	18.2	17.8	0.002	0.96
Elevated aldosterone (%)^*^	16.7	29.6	0.774	0.379
Elevated CRP (%)^*^	25	28.6	0.111	0.739
LVH (%)^*^	69.6	52.8	2.226	0.136
ST-T change (%)^*^	8	17.7	1.726	0.189
Left ventricular high voltage (%)^*^	16	15.2	0.009	0.923

BMI, body mass index; SUc, serum uric acid; BUN, blood urea nitrogen; SCr, serum creatinine; LDL, low-density lipoprotein; TG, triglycerides; TC, total cholesterol; AST, aspartate aminotransferase; ALT, alanine aminotransferase; CRP, C-reactive protein.

* *These values refer to the percentage of individuals with abnormal results among all individuals from the same group*.

### Left Ventricular Hypertrophy

Among 150 subjects who underwent echocardiography examination, 83 cases had LVH, including 16 with primary hypertension and 67 with secondary hypertension. Inter-group comparisons showed that the proportions of elevated SUc, SCr, BUN, TC, LDL, and ALT were significantly higher in the LVH group than in the control group (*P* < 0.05). Albuminuria, ST-T change in electrocardiogram, and enlargement of the heart shadow in chest X-ray were more common in the LVH group (*P* < 0.05). Logistic regression analysis was performed with nine factors that had shown significant inter-group differences in the univariate analysis. Results showed that elevated SUc was associated with the increased prevalence of LVH (OR = 6.638, 95% CI = 1.349–32.657, *P* = 0.02) (Table 3).

**Table 3:** Factors associated with left ventricular hypertrophy.

**Parameters**	**B**	**SE**	**Wald**	**OR**	**95% CI**	***P*-value**
Albuminuria	−0.186	0.759	0.06	0.83	0.188–3.671	0.806
Elevated SUc	1.893	0.813	5.422	6.638	1.349–32.657	0.020
Elevated BUN	−0.327	1.036	0.1	0.721	0.095–5.49	0.752
Elevated SCr	−0.476	0.953	0.249	0.621	0.096–4.021	0.618
Elevated TC	0.399	0.898	0.198	1.491	0.256–8.674	0.657
Elevated LDL	0.29	0.864	0.112	1.336	0.246–7.272	0.737
Elevated ALT	−0.582	0.78	0.557	0.559	0.121–2.576	0.455
ST-T change	0.589	0.837	0.496	1.802	0.350–9.289	0.481
Enlargement of the heart shadow	1.322	0.737	3.22	3.75	0.885–15.882	0.073

*OR, odds ratio; CI, confidence intervals; BUN, blood urea nitrogen; SUc, serum uric acid; SCr, serum creatinine; LDL, low-density lipoprotein; TC, total cholesterol; ALT, alanine aminotransferase*.

### Hypertensive Retinopathy

Of a total of 67 patients who underwent fundoscopy, 27 subjects showed the fundus lesion. Eight subjects belonged to the primary hypertension group and 19 cases to the secondary hypertension group. A higher proportion of elevated TG was observed in the hypertensive retinopathy group than in the non-hypertensive retinopathy group (*P* = 0.001). There were no marked differences in other evaluated factors between the two groups.

### Hypertensive Encephalopathy and Intracranial Hemorrhage

In the present study, a total of 41 cases (including a single case of primary hypertension and 40 cases of secondary hypertension) were diagnosed with hypertensive encephalopathy. Among them, renal disorders were the most common underlying cause, while less common causes included endocrine disorders, drug-induced hypertension, central nervous system disorder, and primary hypertension. All participants were divided into the hypertensive encephalopathy group and non-hypertensive encephalopathy group. The frequencies of elevated SUc, SCr, BUN, TC, TG, and LDL were higher in the hypertensive encephalopathy group than in the non-hypertensive encephalopathy group (*P* < 0.05). Likewise, albuminuria was more common in the hypertensive encephalopathy group (*P* = 0.005). Logistic regression analysis showed that hypertensive encephalopathy was associated with elevated BUN level (OR = 4.384, 95% CI = 1.148–16.746, *P* = 0.031, Table 4). A total of 100 patients underwent cranial CT/MRI examination, among which 10 cases had intracranial hemorrhage. No significant differences in the laboratory results, sex, and BMI were detected between the intracranial hemorrhage group and non-intracranial hemorrhage group.

**Table 4:** Factors associated with hypertensive encephalopathy.

**Parameters**	**B**	**SE**	**Wald**	**OR**	**95% CI**	***P*-value**
Albuminuria	0.851	0.762	1.247	2.342	0.526–10.425	0.264
Elevated SUc	−0.055	0.539	0.010	0.946	0.329–2.723	0.919
Elevated BUN	1.478	0.684	4.671	4.384	1.148–16.746	0.031
Elevated SCr	−0.13	0.543	0.057	0.878	0.303–2.543	0.811
Elevated TG	−0.004	0.494	0.000	0.996	0.378–2.623	0.993
Elevated TC	1.099	0.685	2.576	3.001	0.784–11.481	0.109
Elevated LDL	−0.268	0.643	0.174	0.765	0.217–2.695	0.676

*OR, odds ratio; CI, confidence intervals; BUN, blood urea nitrogen; SUc, serum uric acid; SCr, serum creatinine; TG, triglycerides; LDL, low-density lipoprotein; TC, total cholesterol*.

## Discussion

Despite an increase in the prevalence of primary hypertension, secondary hypertension remains the leading form of pediatric hypertension (19). Indeed, in the current study, 86.3% of the pediatric subjects had secondary hypertension, which is in agreement with previous studies (20). Our results showed that renal diseases were the most common cause of secondary hypertension, accounting for 59.32% of cases (55.37% with renal parenchymal disease and 3.95% with renal vascular disease). Similarly, a previous retrospective study among 132 hypertensive children showed that renal disorders were the most frequent cause of hypertension, with 67% of the patients showing renal or renovascular disease (21). A similar result was found in a large single-center study in Poland, where renal parenchymal diseases were responsible for hypertension in 68% of cases (22).

Given that the treatment and prognosis differ between primary and secondary hypertension, it is vital to distinguish them. Our study showed that four factors were highly suggestive of secondary hypertension, i.e., elevated SCr level, elevated BUN level, elevated SUc level, and albuminuria, which is consistent with the high prevalence of renal disease in pediatric hypertension. The elevation of SCr level was the strongest predictor of secondary hypertension in this study, as it increased the risk of secondary hypertension by 7.22 times. Similarly, in a retrospective study of 167 patients between 5 and 19 years of age, Baracco et al. (23) found that a higher proportion of patients with secondary hypertension showed abnormal creatinine level compared with patients with primary hypertension. We also showed that elevated BUN level was associated with a 6.33-fold higher prevalence of secondary hypertension. Albuminuria was also a strong predictor of secondary hypertension, which is in agreement with the study by Ye et al. (24) in China. Elevated SUc level was also associated with secondary hypertension in this study, although a previous report showed a correlation between SUc and primary hypertension (25). These differences may reflect differences in the sample characteristics. Indeed, as the majority of patients in our study had renal disease, they were more likely to have abnormal SUc level. Although the Fourth Report on the Diagnosis, Evaluation, and Treatment of High Blood Pressure in Children and Adolescents by National High Blood Pressure Education Program Working Group concluded that children with primary hypertension were often overweight (17), we did not find significant inter-group variations in BMI. The probable reason was that our patients were inpatients, and common drug-induced hypertension, especially due to steroids, may have had an effect on the weight of the patients.

Although cardiovascular events and death are rare in hypertensive pediatric patients, TOD has been detected in children with high BP, which is considered to be a risk factor for future cardiovascular events (26). Accordingly, early identification of TOD might prevent the development of hypertension and later consequences. LVH refers to a pathological remodeling of the heart in response to the raised afterload of the left ventricle, and it is considered the most prominent form of TOD caused by hypertension (17, 27). Our data showed that elevated SUc level was independently associated with LVH. A previous study in 130 patients with primary hypertension showed that the SUc level was higher among the hypertensive patients than in the control group, and the patients with hyperuricemia were more likely to have LVH than those with a normal SUc level (12). This result is compatible with our findings. The link between SUc and LVH may be explained by the dysfunction of endothelial cells and the overactivity of the renin-angiotensin system. SUc appears to have the ability to induce endothelial dysfunction and smooth cell proliferation, which are correlated with cardiac hypertrophy (28–30). By activating the renin-angiotensin system, SUc promotes cardiomyocyte growth as well as water and salt retention, which can lead to myocardial hypertrophy (30).

Hypertensive retinopathy, a series of retinal signs related pathologically to retinal microvascular damage due to high BP, has been regarded as a risk indicator of cardiovascular and cerebrovascular diseases (31, 32). Therefore, it is important to investigate the factors associated with hypertensive retinopathy. In this study, we found that the proportion of patients with elevated TG was significantly higher in the hypertensive retinopathy group than in patients without retinopathy. Similarly, Gupta et al. (33) found a positive association of TG with hypertensive retinopathy in adults. However, the pathogenesis is still unclear, and further studies are needed to ascertain the association of TG and hypertensive retinopathy.

Hypertensive encephalopathy manifests as an acute brain dysfunction (disturbance of consciousness, severe headache, convulsions, and visual impairment) induced by an acute and severe increase in BP, which may be one of the earliest symptoms of hypertension in children (34). Hypertensive encephalopathy is a result of cerebral hyperperfusion or an exceeded upper limit of autoregulation by cerebral vessels, which results in cerebral edema and petechial hemorrhages (35). A previous study demonstrated that hypertensive encephalopathy was more common in pediatric patients with renal disorders (34). Similarly, in the present study, there were 41 subjects diagnosed with hypertensive encephalopathy, among which renal diseases accounted for 82.9% of cases. Univariate analyses showed that the proportions of patients with elevated BUN, SUc, SCr, TC, TG, and LDL were significantly higher in the hypertensive encephalopathy group than in the non-hypertensive encephalopathy group. Albuminuria was more common in the hypertensive encephalopathy group. However, after multivariate analysis in logistic regression, only elevated BUN remained independently associated with hypertensive encephalopathy. A possible explanation for this result may be that BUN makes children more susceptible to autoregulation dysfunction, leading to hypertensive encephalopathy (36).

## Limitations

There were several limitations to this study. This was a single-center retrospective study. Further prospective studies are needed to confirm our conclusions. Moreover, we only reviewed the patients' data during their hospitalization without a long-term follow-up, which may have influenced the results of TOD. Finally, there may be some selection bias owing to the retrospective nature of the study.

## Conlusions

In conclusion, this study showed that hypertension in children was most often secondary, with renal disease (especially renal parenchyma disease) as the leading cause. In addition, elevated levels of SCr, BUN, SUc, and albuminuria were associated with secondary hypertension. Elevated SUc and BUN levels were independently associated with LVH and hypertensive encephalopathy, respectively. Elevated TG level was positively associated with hypertensive retinopathy. The possible main clinical contribution of this study is the identification of clinical factors at admission suggestive of secondary hypertension and TOD. Further prospective studies are needed to definitively establish clinical clues for the detection of hypertension in children.

## Data Availability Statement

The original contributions presented in the study are included in the article/Supplementary Material, further inquiries can be directed to the corresponding author.

## Ethics Statement

This study was approved by the ethics committee of the First Affiliated Hospital of Guangxi Medical University (Nanning, China).

## Author Contributions

ZY and YH analyzed the data and drafted the article. They contributed equally to the work and are considered as co-first authors. YQ contributed to the data collection and analysis. YP made contributions to the conception and design of the study and revised the manuscript. All the authors read the final version of manuscript and gave their approval for publication.

## Conflict of Interest

The authors declare that the research was conducted in the absence of any commercial or financial relationships that could be construed as a potential conflict of interest.
