# Supplementary material for: Clinical Characteristics and Factors Associated With Hypertension in 205 Hospitalized Children: A Single-Center Study in Southwest China
Source: Front Pediatr. 2021 Apr 7;9:620158. doi: 10.3389/fped.2021.620158 (PMC8058176; doi:10.3389/fped.2021.620158)
Supplement: Supplementary file 1 [file Table_1.DOCX]

Supplementary materials

Table S1 Causes of secondary hypertension

| Causes | Numbers | Proportion (%) |
| --- | --- | --- |
| Renal disorders | 105 | 59.32 |
| Renal parenchymal disease | 98 | 55.37 |
| Glomerulonephritis | 8 | 4.52 |
| Nephrotic syndrome | 15 | 8.47 |
| IgA nephropathy | 4 | 2.26 |
| Lupus nephritis | 41 | 23.16 |
| Purpura nephritis | 3 | 1.69 |
| Chronic kidney disease | 5 | 2.82 |
| Acute renal injury | 1 | 0.56 |
| Polycystic kidney disease | 2 | 1.13 |
| Hydronephrosis | 4 | 2.26 |
| Renal calculosis | 1 | 0.56 |
| Renal neoplasms | 3 | 1.69 |
| Adrenal tumor | 4 | 2.26 |
| Kidney involvement in other diseases | 7 | 3.95 |
| Renovascular disease | 7 | 3.95 |
| Renal artery stenosis | 6 | 3.39 |
| Renal artery dissection | 1 | 0.56 |
| Drug-induced hypertension | 53 | 29.94 |
| Cardiovascular disease | 6 | 3.39 |
| Coarctation of the aorta | 5 | 2.82 |
| Interruption of the aortic arch | 1 | 0.56 |
| Endocrine disorders | 6 | 3.39 |
| Adrenocortical hyperfunction | 1 | 0.56 |
| 17,20-lyase deficiency | 1 | 0.56 |
| Primary aldosteronism | 1 | 0.56 |
| Hypercorticism | 1 | 0.56 |
| Congenital hyperammonemia | 1 | 0.56 |
| Pheochromocytoma | 1 | 0.56 |
| Nervous system disorders | 6 | 3.39 |
| Viral encephalitis | 3 | 1.69 |
| Craniocerebral trauma | 1 | 0.56 |
| Guillain–Barre syndrome | 1 | 0.56 |
| Neuroblastoma | 1 | 0.56 |
| Unclassified cause | 1 | 0.56 |
